# Prophylaxis for the Unexpected: An Unusual Case of Herpes B Coverage for an Orangutan Bite in an Emergency Department Patient

**DOI:** 10.7759/cureus.46857

**Published:** 2023-10-11

**Authors:** Tayyab Shakoor, Rachel Grider Cook, Melody L Milliron

**Affiliations:** 1 Emergency Medicine, Lake Erie College of Osteopathic Medicine, Erie, USA; 2 Emergency Medicine, Allegheny Health Network- Saint Vincent Hospital, Erie, USA

**Keywords:** trauma, wounds and fractures, herpes b, bites, orangutan

## Abstract

Patients presenting to the emergency department (ED) with an orangutan bite require a series of prophylactic treatments. We present a unique case of an orangutan bite in a 29-year-old male ED patient who was brought by Emergency Medical Services (EMS) for evaluation of an orangutan bite to the right upper extremity with a suspected fourth proximal phalanx fracture. He was referred to hand surgery for a washout and open reduction. Upon discharge, prophylactic medical treatments for unusual pathologies such as herpes B were considered. Appropriate evaluation and treatment of this unique ED condition is necessary to prevent additional patient morbidity.

## Introduction

Animal bites pose a complex meshwork of problems clinically, and major sources of morbidity include secondary infections, soft tissue injuries, and rabies exposure [1]. Monkey bites account for the second most prevalent animal bite injury in the world, at up to 21% of animal bite injuries annually [1]. Treatment varies by animal type, bite location, clinical findings, and the underlying health status of the patient [1]. The sequence of treatments includes immediate wound cleansing with soap and water, prophylactic antibiotics, administration of tetanus vaccination as needed, and depending on the animal’s vaccination status, rabies post-exposure treatment, and potential herpes B vaccination [1]. Herpes B virus is caused by the Macacine herpesvirus 1, which is related to the common double-stranded DNA virus herpes simplex virus (HSV). Transmitted through bites, secretions, and scratches by populations of monkeys, herpes B can present with vesicular lesions, flu-like symptoms, respiratory failure, diplopia, altered level of consciousness, and even death [2]. Herpes B virus can be transmitted by monkey bites. Therefore, monkey bites should be treated with 14-day prophylactic antiviral therapy with valacyclovir [3].

## Case presentation

A 29-year-old male presented to the emergency department (ED) with a chief complaint of multiple animal bites to the right upper extremity from an orangutan. The orangutan bit the right hand and forearm of the patient and pulled it through a mesh screen. The injury also resulted in dislocation of the patient’s fourth finger. Emergency Medical Services (EMS) subsequently arrived on the scene and applied pressure to stop the bleeding. There were no additional traumatic injuries seen. The patient’s past medical and surgical history were not contributory. Initial vital signs were a heart rate of 94, afebrile, blood pressure 148/107, and a respiratory rate of 22 with 100% oxygen saturation on room air.

**Figure 1:**
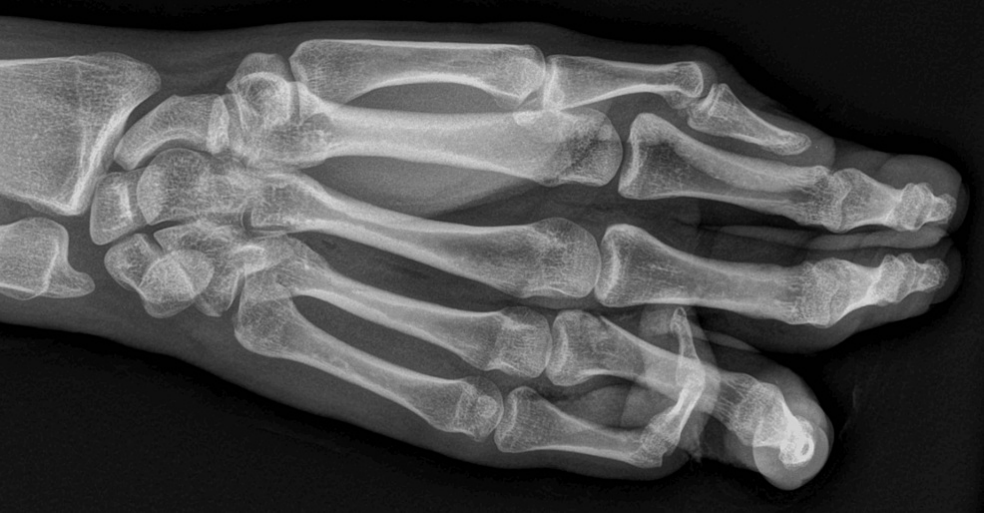
Anterior-posterior radiograph of the hand shows a transverse metaphyseal fracture at the base of the 4th proximal phalanx with dorsal angulation. The fifth digit is uninjured.

The physical examination revealed the right shoulder to be unremarkable, whereas there was tenderness of the right elbow over the lateral epicondyle. Several lacerations consistent with bite wounds were noted with associated crepitus upon palpation alongside multiple puncture wounds extending from the right forearm to the elbow. Also, an obvious deformity of the 4th right finger at the medial aspect of the distal interphalangeal (DIP) prompted concern for an open fracture. Consequently, an X-ray was ordered and showed a transverse metaphyseal fracture to the base of the 4th proximal phalanx with dorsal angulation (Figure 1). Moreover, a CT showed no evidence of an additional injury to the right upper extremity; however, an open fracture of the base of the right 4th proximal phalanx was confirmed.

The ED physician recalled unusual exposures involving orangutan bites and called the on-call zoo veterinarian to inquire about the orangutan’s health status, especially simian immunodeficiency viruses (SIV). The orangutan was known negative for SIV, and the ED physician called the on-call infectious disease physician and informed them that the animal had not been checked previously for herpes B. As a result, the patient’s initial treatment regimen included morphine for pain management, valacyclovir, Unasyn, rabies vaccination, and immunoglobulin.

The patient was taken to the operating room (OR) for washout and open reduction of the proximal 4th phalanx fracture. The dislocation was previously reduced in the ED. No additional injuries were noted. Additional wounds were left to heal by secondary intent. At discharge, the physician’s orders included a continuation of the rabies vaccination series, amoxicillin/clavulanic acid, and valacyclovir prophylaxis for 14 days. The patient was further instructed to follow up and watch for signs and symptoms of herpes B infection for 5 weeks in layman’s terms: vesicular lesions, tingling, pain, or itching at the exposure site, lymphadenopathy, flu-like illness, hepatitis, pneumonitis, neurologic symptoms, and respiratory failure.

## Discussion

Additional areas for concern in a patient with an orangutan bite include rabies exposure, bleeding risk, and possible fractures. Diagnosis is based mainly on clinical signs and symptoms, patient history, and physical exam. One additional consideration in monkey bites is knowledge of the animal’s health status and prior serological history of SIV. Biologically similar to human immunodeficiency virus (HIV), SIV is documented to have crossed from nonhuman primates to humans in April of 1990 [4]. Although clinical data is largely unknown in humans, and isolating and diagnosing SIV is difficult, proper screening of primates involved in bites that are seropositive for SIV should be a consideration, albeit negative in our orangutan.

Herpes B is an infrequent viral pathology in animal bites and prophylaxis is largely unique to monkey bites. Herpes B requires laboratory testing, such as serological testing for antibodies, or polymerase chain reaction (PCR) if symptoms present in the patient or from the associated monkey [5]. Patient signs and symptoms of active herpes B infection include flu-like symptoms, vesicular lesions, difficulty breathing, neurological effects such as confusion, dysphagia, coma, and eventual death [2, 6]. In addition to the usual treatments with tetanus vaccination, amoxicillin/ clavulanic acid, herpes B prevention with valacyclovir was prescribed at discharge. The need for valacyclovir treatment is important as signs and symptoms of herpes B present within 48 hours of the onset of the animal bite [2]. Current recommendations require valacyclovir three times daily for two weeks. Apart from an open reduction of the patient’s 4th proximal phalanx, the rest of the wounds were left to heal by secondary intention.

## Conclusions

Orangutan bites require unique consideration for herpes B prevention with valacyclovir. If untreated, late signs and symptoms of herpes B may include vesicular lesions, tingling, pain, and itching at the exposure site, lymphadenopathy, flu-like illness, hepatitis, pneumonitis, neurologic symptoms, respiratory failure, and death. Additionally, orangutan bite treatment includes tetanus vaccination, amoxicillin/clavulanic acid to prevent bacterial infection, and rabies vaccination consideration. Overall, good trauma care and appropriate prophylaxis should reduce the risk of morbidity from monkey bites.

## References

[1] Cohen JI, Davenport DS, Stewart JA, Deitchman S, Hilliard JK, Chapman LE, B Virus Working Group (2002). Recommendations for prevention of and therapy for exposure to B virus (cercopithecine herpesvirus 1). Clin Infect Dis.

[2] (2022). World Health Organization Animal Bites. https://www.who.int/news-room/fact-sheets/detail/animal-bites.

[3] Centers for Disease Control and Prevention. (2019, January 31) (2022). Centers for Disease Control and Prevention: Herpes B virus: Information for Healthcare Providers. https://www.cdc.gov/herpesbvirus/healthcare-providers.html.

[4] Khabbaz RF, Heneine W, George JR (1994). Infection of a laboratory worker with simian immunodeficiency virus. N Engl J Med.

[5] Centers for Disease Control and Prevention (2022). Centers for Disease Control and Prevention: Laboratory Testing and Diagnosis for Herpes B Virus. https://www.cdc.gov/herpesbvirus/laboratory.html.

[6] Riesland NJ, Wilde H (2015). Expert review of evidence bases for managing monkey bites in travelers. J Travel Med.

